# Efficacy and safety of Shi-style cervical manipulation therapy for treating acute and subacute neck pain: study protocol for a randomized controlled trial

**DOI:** 10.1186/s13063-021-05062-6

**Published:** 2021-02-08

**Authors:** Mingcai Zhang, Guoqing Du, Congying Liu, Wei Li, Jiayu Yang, Bo Chen, Xiaoyue Yu, Yizhe Xiong, Enyu Jiang, Ningyang Gao, Sumin Jiang, Zhenqiu Xu, Xiang Wang, Hongsheng Zhan

**Affiliations:** 1 Shi’s Center of Orthopedics and Traumatology, Shuguang Hospital Affiliated to Shanghai University of TCM, Institute of Traumatology & Orthopedics, Shanghai Academy of TCM, Shanghai, 201203 People’s Republic of China; 2 Shanghai University of TCM, Shanghai, People’s Republic of China; 3 Jing’an District Central Hospital of Shanghai, Shanghai, People’s Republic of China; 4 Xiangshan TCM Hospital, Huangpu District, Shanghai, People’s Republic of China

**Keywords:** Acute neck pain, Subacute neck pain, Shi’s cervical manipulation, Effectiveness, Safety, Diclofenac sodium sustained-release capsules

## Abstract

**Background:**

Neck pain is a common clinical disease, which seriously affects people’s mental health and quality of life and results in loss of social productivity. Improving neck pain’s curative effect and reducing its recurrence rate are major medical problems. Shi’s manipulation therapy has unique advantages and technical features that aid in the diagnosis and treatment of neck pain. Compared with first-line non-steroidal anti-inflammatory drug (NSAID) treatment of neck pain, Shi’s cervical manipulation lacks the relevant research basis of therapeutic advantage, safety, and satisfaction for treating acute and subacute neck pain. Herein, we aim to confirm our hypothesis in a clinical trial that the safety and efficacy of Shi’s cervical manipulation will be more effective, safer, and more satisfactory than NSAIDs to treat acute and subacute neck pain.

**Methods:**

In this multicenter, positive-controlled, randomized clinical trial, traditional analgesic drug (NSAID) is used to evaluate and show that Shi’s manipulation is more effective, safe, and satisfactory for treating acute and subacute neck pain. Overall, 240 subjects are randomly divided into the trial and control groups, with both groups treated by the corresponding main intervention method for up to 12 weeks. Clinical data will be collected before the intervention and immediately after the first treatment; at 3 days and 1, 2, 4, 8, and 12 weeks after the intervention; and at 26 and 52 weeks after treatment follow-up of clinical observation index data collection. The clinical observation indices are as follows: (1) cervical pain is the primary observation index, measured by Numerical Rating Scale. The secondary indices include the following: (2) cervical dysfunction index, measured by patient self-evaluation using cervical Neck Disability Index; (3) cervical activity measurement, measured by the cervical vertebra mobility measurement program of Android mobile phone system; (4) overall improvement, measured by patient self-evaluation with SF-36; and (5) satisfactory treatment, determined by patient self-evaluation.

**Discussion:**

We will discuss whether Shi’s cervical manipulation has greater advantages in efficacy, safety, and satisfaction of acute and subacute neck pain than traditional NSAIDs, to provide a scientific basis for the dissemination and application of Shi’s cervical manipulation.

**Trial registration:**

China Registered Clinical Trial Registration Center ChiCTR1900021371. Registered on 17 February 2019

## Introduction

### Background and rationale {6a}

According to the latest surveys, 71.5% of the general population had neck pain for more than a year [1–3]. Although neck pain caused only 1.7–11.5% of disability, it is worth noting that, equivalent to back pain, it caused a huge burden and disability rate on individuals and the social economy and was listed as 1 of 5 chronic and refractory diseases by the US government. Previous studies confirmed that acute and subacute neck pain is particularly important in the prevention and treatment of neck pain [4, 5]. Patients with acute and subacute neck pain rank significantly lower than healthy people in both mental health and quality of life. In addition, acute and subacute neck pain results in reduced social productivity caused by loss of life function and labor force involvement, and the additional financial burden continues to increase dramatically [6, 7]. How to treat acute and subacute neck pain, improve its curative effect, and reduce recurrence rate are major medical problems faced by people all over the world.

Traditional Chinese medicine (TCM) manipulation therapy has unique advantages and characteristics in the diagnosis and treatment of neck pain. As early as the Qing Dynasty, detailed manual treatment of neck pain was recorded in the Traditional Chinese Medical Orthopedics monograph, *Chinese Bone-setting Diagram*: “If the neck is injured, the head on the back cannot be lowered, or the tendon is long and bone is wrong, or the tendon is gathered, or the tendon is strong, use the second manipulation of *Xiong Gu Zi’s* technique to lift and correct the neck.” On this basis, doctors developed many cervical vertebra techniques and formed different schools with different characteristics and positive curative effects in the diagnosis and treatment of neck pain. “Shi’s Traumatology,” a valuable cultural heritage passed from generation to generation in Shanghai over a hundred of years, is one of the most distinctive schools of “Shanghai culture.” And the cervical vertebra correction technique developed from it, “Dislocation of Bone and Malposition of Ligament,” is mature and effective. Shi’s cervical correction technology can reduce the recurrence rate, improve the efficiency of the price ratio, and support obvious clinical advantages in the diagnosis and treatment of neck pain [8–10]. However, compared with the first-NSAIDs treatment of neck pain, Shi’s cervical manipulation lacks the relevant research basis in terms of efficacy advantage, safety, and satisfaction in the treatment of neck pain.

### Objective {7}

Here, we present the protocol of a multicenter, positive-controlled, randomized clinical study investigating the efficacy and safety of Shi-style cervical manipulation therapy for treating acute and subacute neck pain. Our hypothesis is that Shi’s cervical manipulation would have more advantages in efficacy, safety, and satisfaction to treat acute and subacute neck pain than traditional NSAIDs.

### Trial design {8}

In this study, a multicenter positive-controlled, randomized clinical trial design is used. Patients with acute and subacute neck pain in accordance with the test and research standards are randomly divided evenly into two groups: trial group (with Shi’s manipulation of the cervical spine as the main intervention method) and control group (with oral administration of diclofenac as the main intervention method), with both groups treated by corresponding main intervention method for up to 12 weeks. Clinical data will be collected before the intervention and immediately after the first treatment; at 3 days and 1, 2, 4, 8, and 12 weeks after the intervention; and at 26 and 52 weeks after treatment follow-up of clinical observation index data collection.

## Methods: participants, interventions, and outcomes

### Study setting {9}

This is an exploratory, multicenter, positive-controlled, randomized clinical study. This trial began in October 2019 and ends in October 2021. It is organized and implemented by the Department of Orthopedics and Traumatology of Shuguang Hospital Affiliated with Shanghai University of Traditional Chinese Medicine. The research is planned to be completed in Shuguang Hospital Affiliated with Shanghai University of Traditional Chinese Medicine (Shuguang Hospital for short), Shanghai Jing’an District Central Hospital (Jing’an Center Hospital for short), and Shanghai Xiangshan Hospital of Traditional Chinese Medicine (Xiangshan Hospital for short). Shuguang Hospital is a hundred-year-old hospital in Shanghai. It is a grade III, class A comprehensive hospital of traditional Chinese medicine. It is a national model hospital of traditional Chinese medicine and has three key disciplines of the Ministry of Education. Shi’s orthopedics and traumatology, as one of the key disciplines of the Ministry of Education, has a history of nearly 150 years and is one of the first national intangible cultural hospitals. Jing’an Central Hospital is a grade III, class B general hospital with a history of more than 70 years. It is the teaching hospital of Shanghai Second Medical University, Shanghai University of Traditional Chinese Medicine, and Medical College of Shanghai Tongji University. Xiangshan Hospital is one of the first grade II, class A hospitals of traditional Chinese medicine in Shanghai. It has a history of more than 40 years, which is a hospital of traditional Chinese medicine and integrated traditional Chinese and Western medicine. All three hospitals are responsible for the program’s therapeutic interventions and are located in Shanghai, China, and Shuguang Hospital is the main responsible center of this project. The reasons for choosing these three hospitals as the research centers of this project are as follows, On the one hand, these three hospitals are all located in the center of Shanghai, with dense population and convenient transportation, which is conducive to the treatment and follow-up of the subjects; on the other hand, the orthopedics and traumatology department of these three hospitals is very famous in Shanghai, and Shi’s orthopedics and traumatology department of Shuguang Hospital and the other two hospitals often carry out cooperation in clinical diagnosis and treatment techniques and scientific research. These convenient conditions contribute to the smooth development of this research project. According to the standard implementation method of randomized multicenter clinical trials, patients with neck pain who meet the study standard are randomly divided into trial group and control group (subjects 1:1).

In the trial group, we used Shi’s cervical manipulation as the main intervention method. Shi’s manipulation intervention scheme was pre-tested in the experimental study of our research group. It was effective immediately after the shortest treatment time, and the longest treatment time was 12 weeks.

In the control group, we used oral administration of diclofenac sodium sustained-release capsules (Difene, manufacturer Temmler Ireland, Ltd.; 75 mg), the first-line non-steroidal anti-inflammatory drug to treat neck pain reported in the literature as the main intervention [11]. Clinical data collection will be completed in the Department of Orthopedics and Traumatology outpatient centers of Shuguang Hospital, Jing’an Center Hospital, and Xiangshan Hospital.

### Eligibility criteria {9}

**Fig. 1:**
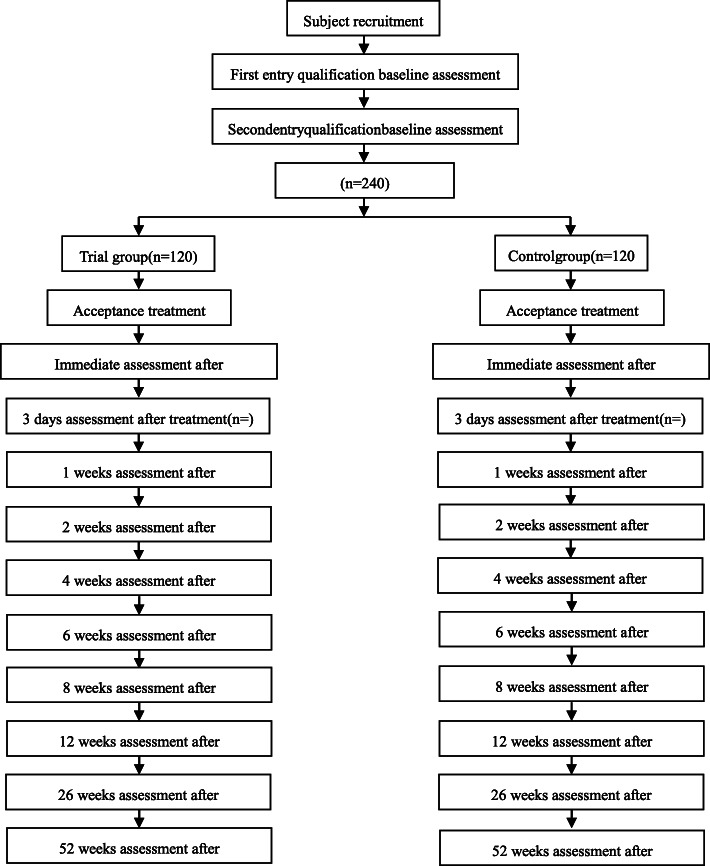
Research flow chart

The subjects of each research center participating in this study shall first meet the following diagnostic standards of acute and subacute neck pain [12, 13] and then determine eligibility according to the inclusion and exclusion criteria. The subjects included in this study shall carry out the follow-up study in strict accordance with the exclusion and removal criteria, and the specific operation route is shown in Fig. 1.

### Diagnostic criteria for acute and subacute neck pain [12, 13]

1. Primary symptom of mechanical, non-specific neck pain symptoms, with a duration of < 12 weeks
2. Pathologic criteria and degree criteria for neck pain: neck pain equivalent to grade I or II according to the Bone and Joint Decade 2000–2010 Task Force on Neck Pain and Its Associated Disorders classification (4 levels in total) [13];

Grade I neck pain: No signs or symptoms suggestive of major structural pathology, and no or minor interference with activities of daily living; will likely respond to minimal intervention such as reassurance and pain control; does not require intensive investigations or ongoing treatment.

Grade II neck pain: No signs or symptoms of major structural pathology, but major interference with activities of daily living; requires pain relief and early activation/intervention aimed at preventing long-term disability.

### Inclusion criteria

1. Age range from 18 to 65 years.
2. The subjects meet the above diagnostic criteria for acute and subacute neck pain.
3. NRS score of ≥ 3.
4. It is an axial (non-root) neck pain caused by changes in the facet joints, discs, muscles, and ligaments [1].
5. Those who are willing to participate in this experiment and sign the consent letter.

### Exclusion criteria

1. Cervical spine fracture and dislocation
2. Cervical vertebra, cervical soft tissue, cervical spinal cord tumor, and tuberculosis.
3. Cervical spine fusion, paravertebral bridge, and severe osteoporosis
4. A history of cervical spine surgery
5. A history of severe trauma to the cervical spine or neck
6. Neck with skin inflammation, skin damage, etc.
7. Patients with serious heart, liver, kidney, and hematopoietic system diseases; digestive system ulcer; bleeding; black stool; other diseases; and mental illness
8. Patients with extreme physical deficiency and pregnant women
9. Participants who participated in clinical trials of other drugs and treatments within the previous 3 months that would interfere with this study
10. Meet grade III or IV according to the Bone and Joint Decade 2000–2010 Task Force on Neck Pain and Its Associated Disorders classification

Grade III neck pain: No signs or symptoms of major structural pathology, but the presence of neurologic signs such as decreased deep tendon reflexes, weakness, and/or sensory deficits might require investigation and occasionally more invasive treatments.

Grade IV neck pain: Signs or symptoms of major structural pathology, such as fracture myelopathy, neoplasm, or systemic disease; requires prompt investigation and treatment.

Those who meet 1 of the above conditions cannot be included in this study.

### Rejection criteria

1. Patients misdiagnosed as acute subacute neck pain
2. The subjects mistakenly included (meeting the exclusion criteria)
3. Those who did not receive treatment strictly and used drugs according to the regulations after being selected
4. No detailed treatment record
5. Those who disobeyed the trial plan and combined with other drugs
6. Those who still received other related treatment that affected the evaluation of this study at the time of selection and could not stop

Those who meet 1 of the above conditions should be rejected.

### Shedding criteria

1. Intolerable adverse reactions.
2. Serious adverse reactions.
3. The patients’ pain continued to increase, which proved that trial participation was not suitable.
4. The patient’s health may be damaged (for example, serious complications).
5. Those who voluntarily withdraw or miss visits in the middle.

Those who meet 1 of the above conditions should be shedding cases.

### Participants {26b}

Information and informed consent forms have been prepared in accordance with the guidelines of the China registered clinical trial ethics review committee. Potential participants receive both forms at least 1 day before their screening visit. During this visit, a study physician explains all study procedures, and written informed consent is only given after participants had adequate time to ask questions. We include male and female subjects with acute and subacute neck pain in this study. Each subject should voluntarily sign an informed consent form before testing begins in the study.

### Interventions

#### Intervention description {11a}

##### Trial group

The main intervention method in the trial group is Shi’s cervical manipulation therapy. The manipulation treatment team is composed of six clinicians (two in each of the three trial centers) with a minimum of 5 years’ experience in manual operation, who are good at manual spine diagnosis and treatment and have received standard operating procedures (SOP) training in this study. Each selected subject should have a 3-dimensional (3D) computed tomography (CT) reconstruction of the cervical vertebra, as well as a special physical examination of the cervical vertebra. Each subject will receive an initial diagnosis for 15–20 min after entering the group, including brief history collection, cervical static palpation (tenderness point and muscle spasm), cervical dynamic palpation (intervertebral looseness of the cervical spine), cervical sequence activity inspection, and cervical nerve reflex. Both history and examination results should be recorded in the case report form. According to the examination results, manipulation clinicians will give a comprehensive diagnostic analysis and manipulation therapy to each subject’s cervical spine. Key points of manipulation therapy operation are as follows: (1) acupoint pressing analgesia operation, according to the treatment principle of local acupoint selection and remote acupoint selection of cervical vertebra; point rubbing; and pressing operation are performed on the thumb pulp. The pressure can be light to heavy, then heavy to light, and the operation is repeated. The specific strength is to press until thumb nail color is white, while considering patient tolerance. Each acupoint is operated for 1 min. The specific acupoints are as follows: (a) local acupoints—Fengchi (International acupoint code GB20), Fengfu (International acupoint code DU16), Tianzhu (International acupoint code Bl10), Wangu (International acupoint code GB12), Dazhui (International acupoint code DU14), Jianjing (International acupoint code GB21), Jianzhongshu (International acupoint code SI15), Quepen (International acupoint code ST12), Tianzong (International acupoint code Si11), and Ashi; (b) remote acupoints—Lieque (International acupoint code LU7), Houxi (International acupoint code SI3), and Hegu (International acupoint code LI4). (2) Bone-setting manipulation operation, according to the results of palpation and 3D CT reconstruction of the patient’s cervical spine, the clinicians will give directional and fixed-point bone-setting manipulation to treat the cervical vertebrae semidislocation. The specific operation is as follows: the patient sits on the treatment chair, with the head slightly bent forward, and the manual operation physician stands at the patient’s side, with the right side as the patient’s side, for example, the manual operation physician locks his left thumb against vertebral plate between the transverse process and the spinous process of the cervical vertebra with semidislocation, and the other four fingers stick to the patient’s left head and neck, and the middle and upper part of the elbow of the right arm slightly bent on the patient’s lower jaw, according to the vertebrae semidislocation direction, the left thumb with pushing force to the vertebral plate in the opposite direction of semidislocation, while the right forearm rotates rapidly to lift and rotate the head with a direction- and size-controlled flashing force. There is a sliding motion and snapping sound from the vertebra under the left thumb. The whole manual operation treatment lasts for 20 min each time, and is done twice a week, with the longest course of treatment lasting for 12 weeks.

##### Control group

The main intervention method in the trial group is oral NSAIDs. Each selected subject should have a 3D CT reconstruction of the cervical vertebra, as well as a special physical examination of the cervical vertebra. The treatment team is composed of three clinicians (one in each of the three trial centers) with the qualification of clinical medical registered doctors and senior professional titles, who have received SOP training in this study. After exclusion of the contraindications of diclofenac sodium (known to be allergic to other components of diclofenac sodium, acetylsalicylic acid, ibuprofen, and Daphne, gastric and duodenal ulcer, gastrointestinal inflammatory disease, black stool, or unknown blood history), the NSAID diclofenac sodium double-release enteric-coated capsules (Difene, manufacturer Temmler Ireland. Ltd.; 75 mg, once a day, once a time, oral) is the main intervention. Each subject will receive an initial diagnosis for 15–20 min after entering the group, including brief history collection, cervical static palpation (tenderness point and muscle spasm), cervical dynamic palpation (intervertebral looseness of the cervical spine), cervical sequence activity inspection, and cervical nerve reflex. Both history and examination results should be recorded in case report form. Each subject will obtain a patient log card, which shall be kept by the patients themselves (handed over to the physician in charge of the group at the end of the test), and will be used to record name, dose, time, discomfort, combined medication, and other information about the drug taken truthfully every day. The specific drug dosage shall be specified by the physician in charge of the group according to the drug use instructions, and the maximum duration of the medication shall be 12 weeks.

#### Criteria for discontinuing or modifying allocated interventions {11b}

Participants may request to leave the study or they may be withdrawn due to study-related adverse events and shedding criteria. If a subject is discontinued from study participation due to an adverse event, they will be evaluated by the study clinicians for the need of additional treatment for neck pain. Safety data will be collected on any subject who is withdrawn from the study. Participants in both study groups may receive additional treatment. Emergency treatment for patients who have severe pain and cannot tolerate manipulation therapy or cannot improve their pain within a short period of time affecting their sleep or life should be given emergency analgesic or sedative, muscle relaxant drugs, and then be rejected from the trial.

#### Strategies to improve adherence to interventions {11c}

To ensure retention of participants, follow-up visits will be scheduled to coincide with routine clinic appointments as far as possible. Moreover, we attract the participants through regular biweekly TCM health lectures in each clinical trial center. In addition, study staff will contact participants, either over the phone or WeChat, before their scheduled follow-up appointment. Finally, participants will receive reimbursement for their time and transportation in the form of a gift card.

#### Relevant concomitant care permitted or prohibited during the trial {11d}

In principle, other pain killers, muscle relaxants, and other drugs are not allowed to be used during the trial. If there is any special situation, it must be used, and the use situation needs to be truthfully recorded in the medication registration form. The patients with other diseases before enrollment will continue to maintain the original treatment after enrollment, and the medication related to pain shall be recorded in the medication record form. When the case falls off or the patient’s compliance is poor, the reasons for the fall off and poor compliance shall be entered in the case observation form in detail, and the patient’s understanding and support shall be obtained by contacting the patient as frequently as possible; the evaluation items that can be completed shall be completed, and the last treatment time shall be recorded. The case fall off, the patient’s compliance, and adverse reactions shall be statistically described and compared and evaluated between the groups.

#### Provisions for post-trial care {30}

Once participants complete the study, they will be able to continue receiving clinical care from respective clinical trial centers, for example, TaiChi, Baduanjin, characteristic cervical function training guidance techniques of TCM. Participants study records will be reviewed if necessary. For the patients who have completed the trial but have no effect on remission, doctors should provide alternative treatment, such as combined traction, acupuncture, and other comprehensive conservative treatment, as well as the application of analgesics and muscle relaxants. When the sleep patterns and quality of life of the patients are seriously affected, patients can be identified as inpatients for further treatment, including nerve block injection, surgery, and other treatments.

#### Outcomes {12}

##### Effectiveness evaluation index

The manual intervention program was pre-tested in our research group’s experimental study and was in effect immediately after the shortest treatment, and the longest treatment time was 12 weeks. Combined with the reported literature on related neck pain [14, 15], both groups are treated according to the main intervention methods of each group for a period of up to 12 weeks. Clinical data will be collected before the intervention and immediately after the first treatment; at 3 days and 1, 2, 4, 8, and 12 weeks after the intervention; and at 26 and 52 weeks after treatment follow-up of clinical observation index data collection. The clinical observation indexes are as follows: (1) cervical pain is the primary observation index, measured by NRS [16] and composed by a horizontal line with a length of 0–10 cm, and the anchor words are “0” for “no pain” and “10” for “extreme pain.” Participants are asked to select a point on the NRS line segment that corresponds to their current or recent week’s average pain intensity and draw a vertical line as a marker to express the pain intensity. The researchers determine the NRS score by measuring the distance from the starting point of “painless” to the marker point; the higher the score is, the stronger the degree of pain is. The specific judgment is as follows: 1–3 cm is mild pain, 4–6 cm is moderate pain, and 7–10 cm is severe pain. The secondary indices include the following: (2) cervical dysfunction index, measured by patient self-evaluation with cervical NDI [17, 18], which has 10 items in total, six options under each item, from light to heavy, corresponding to the score of 0–5, the lowest score of each item is 0, and the highest score is 5; the higher the score is, the more serious the degree of dysfunctions. NDI (%) = [(total score)/(number of items completed by subjects × 5)] × 100. The specific judgment is as follows: 0–20% indicates mild dysfunction, 21–40% indicates moderate dysfunction, 41–60% indicates severe dysfunction, 61–80% indicates extremely severe dysfunction, and 81–100% indicates complete dysfunction (whether subjects have exaggerated symptoms should be checked in detail); (3) cervical activity measurement [19], measured by the cervical vertebra mobility measurement program of Android mobile phone system, with a minimum of 5 degrees carry measurement; (4) overall improvement, measured by self-evaluation of patients with SF-36 (The Medical Outcomes Study 36-Item Short-Form Health Survey) [20, 21], which is composed of physical functioning, role-physical, bodily pain, general health, vitality (VT), social functioning (SF), role-emotional, mental health, and health transition; and (5) satisfactory treatment, determined by patient self-evaluation, is 1 of 3 levels: dissatisfied, satisfied, and very satisfied.

##### Safety evaluation index

During the test, we should observe whether there are adverse reactions and adverse events, and make records. If there are adverse events, such as adverse effects on patients’ consciousness, feeling, exercise, sleep, blood pressure, pulse, heart rate, respiration, and other normal physiological indicators, cervical fracture, skin damage, spinal cord and vertebral artery injury, gastrointestinal discomfort or ulcer, bleeding, and black stool, the occurrence of adverse reactions shall be recorded in detail on the case observation form, including the time, severity, duration, and treatment measures, and correlation with experimental treatment method shall be analyzed with comprehensive consideration of complications and combined treatment. In case of adverse reactions, the clinical observation physician can decide whether to stop the trial according to the patient condition. Patients who stop treatment due to adverse reactions should be tracked and investigated, and the results should be recorded in detail.

#### Participant timeline {13}

**Fig. 2:**
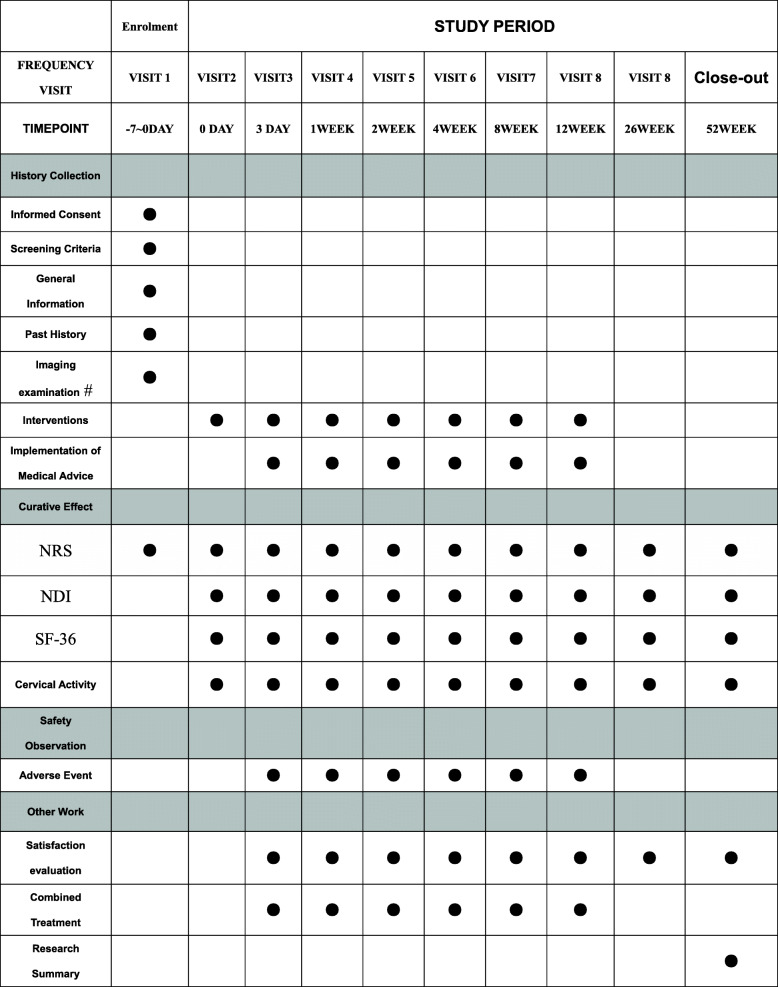
Recommended content for the schedule of enrolment, interventions, and assessments. ^#^If the subject has had the relevant imaging examination within 3 months and can provide the report form, no additional imaging examination is required. *If the subjects were directly enrolled at the first visit, visit 1 and visit 2 coincided in time, and visit 2 index was taken as the baseline

Participants in both groups are treated according to the main intervention methods of each group for a period of up to 12 weeks. Clinical data are collected before the intervention and immediately after the first treatment; at 3 days and 1, 2, 4, 8, and 12 weeks after the intervention; and at 26 and 52 weeks after treatment follow-up of clinical observation index data collection (see Fig. 1 for the participant timeline for the trial and Fig. 2 for the study assessments).

#### Sample size {14}

In this project, the trial group and the control group are to be set up. This study is designed with the control group as the optimal design, and the results are summarized according to previous clinical trials and literature [2]. According to the results of previous clinical trials and literature summary, the first observation index after 12 weeks of treatment was NRS score of neck pain, 1.50 ± 1.70 in the trial group, 2.08 ± 1.65 in the control group; *α* = 0.05 (bilateral test), *β* = 0.10, boundary value 0.08, sample size ratio *k* = 1 in both groups. The PASS software is used to calculate the sample size of 102 cases in the two groups. Considering that the sample size loss rate is about 10–20%, further calculated the sample size of each group is *N* = 120.

#### Subject recruitment {15}

According to the sample size requirements of 120 cases in the trial group and the control group, this project uses written and online media promotion, broadcast, and other ways to recruit subjects with neck pain to visit Orthopedic Department Outpatients of Shuguang Hospital, Jingzhong Hospital, and Xiangshan Hospital according to the principles of convenient transportation and proximity. The subjects need to go through two baseline diagnostic screenings by clinicians in each research center. The clinicians in charge of diagnosis and screening are not aware of the random grouping. During the scheduled clinical visit, the study clinicians will provide a brief overview of the study. If the participant is interested, the clinicians will determine patient eligibility according to the above eligibility criteria. If the patient is eligible and agrees to participate, the clinicians will conduct the informed consent and enrollment. Participants will be able to discuss study details with the clinicians and ask questions before signing the informed consent. Following enrollment, participants will be randomized by the assistants, who are full-time research assistant in charge of patient grouping, accept their cervical spine examination and imaging examination, and receive the assigned treatment.

#### Assignment of interventions: randomization

##### Sequence generation {16a}

In this project, a multicenter positive-controlled, randomized clinical trial design is adopted, and subjects who meet the test and research criteria are randomly divided into two groups (proportion of 1:1), which are the trial group and the control group. The random number table was generated by special statistical staff at Shanghai University of T.C.M. According to strict random procedures by using the SAS software (version 9.4, SAS Company, Cary, NC, USA). The randomized list is stored on a secure database (Microsoft Office Access 2007) by the data manager and is inaccessible by the relevant case observer or other researchers. Each subject can only register and randomize once, and no detailed information about the subject can be deleted from the database.

The design procedures of the specific randomized scheme of multicenter clinical trials include randomized parameter setting, randomized regulations, SAS-randomized program design, and the resulting random numbers, randomized coding of the trial center, randomized coding of the trial cases, and randomized coding of the treatment group.

1. Randomization parameter setting

Seed number, the initial value of the random number, in this study is 151427.

The total number of cases is the sample size. Area group length × area group number = total number of cases: sample size 240 cases, area group length 4, and area group number 60.

Stratification number, taking the center (hospital) participating in the clinical trial as the stratification factor, because the number of cases completed by each participating clinical trial center cannot be determined in advance, the management method of “random center” is adopted in this study; that is, when subjects are enrolled, the person in charge of each participating research center immediately requests the random number and corresponding group code from the “random center” by telephone.

The number of test groups is divided into trial group and control group.

Distribution proportion, the number of samples in each treatment group, is allocated by 1:1.

(2) Randomization rules

Randomization rules for center code distribution

The completion of this study will be done in Shuguang Hospital, Jingzhong Hospital, and Xiangshan Hospital. Each center is sorted into Jing’an J, ShuguangS, and Xiangshan X according to the initials of the hospital abbreviation. Corresponding to the order of “random number of center code distribution” (random number under Center) generated by the SAS statistical software package, the random code of each center is obtained.

Randomization of trial case allocation

This study is divided into two groups: group A is defined as having a Rand of 1, 2, 3, 4, 5, and 6, and group B as having a Rand of 7, 8, 9, 10, 11, and 12.

Randomization rules for distribution of treatment group: this study is divided into two groups. If the first random number is greater than the second random number, group A was the trial group and group B was the control group. Otherwise, group B is the trial group and group A is the control group.

(3) SAS random programming

Using the SAS9.4 unified software, according to the abovementioned random parameters, the software program successively input the seed number corresponding to “proc plan seed,” “random number corresponding to the code of,” title “center,” stratified number corresponding to “factors center” 3; “random number corresponding to the test case of,” title, “factors blocks = 60, Rand = 4,” and other instructions, and then the operation is completed.

##### Concealment mechanism {16b}

In this study, the management method of “random center” is adopted. The random numbers were generated by the staff in charge of project (random center) statistics of Shanghai University of Traditional Chinese Medicine using the SAS software. The heads of clinical research centers, diagnostic intervention physicians, researchers, and participants of symptom and sign clinical data collection are unaware of the random numbers and corresponding groups.

##### Allocation and implementation {16c}

Upon participant enrollment, the diagnostic intervention physician is responsible for asking the person in charge of each participating clinical center for the random number and corresponding group information, and then the person in charge of each participating clinical center immediately asks for the random number and corresponding group code from the “random center” by telephone, and then the person in charge of each clinical center informs the patient of the random number, corresponding group code, and intervention measures by telephone or WeChat. The intervention physician shall not disclose the above information to the subject and the symptom and sign information collection physician.

#### Assignment of interventions: blinding

##### Blind implementation {17a}

The random grouping and intervention plan information is a blind setting for subjects and data statistical analysts. Blinding process: the blinding process is carried out in accordance with the blinding operation specifications of single-blind clinical trial drugs, and a blinding process record file is formed; the blind bottom storage adopts a 2-level blind method: the first level is the code corresponding to two treatment groups (randomly designated as a and b), and the second level is the code corresponding to each code. The two levels of blind bottom are sealed separately, each in duplicate, and stored in the Institute of Orthopedics and Traumatology by full-time personnel (not directly involved in the research work of this project).

##### Procedure for unblinding if needed {17b}

In this study, there are two unblinding provisions. The first unblinding is at the end of blind audit data locking, and the second unblinding is at the end of statistical analysis. According to the emergency unblinding regulations, when patients need to receive emergency treatment due to adverse events, and the emergency treatment is related to the actual situation of receiving the study drug, the project research leader and the project supervisor can decide to open the emergency unblinding and read the corresponding emergency letters. Researchers should fill in the corresponding records with the date, reason, and process of unblinding.

#### Data collection, management, and analysis

##### Data collection {18a}

The data involved in this project mainly include random grouping data, clinical baseline data, intervention symptoms and signs change, follow-up data, and results of statistical analysis data.

The random grouping data shall be provided by the researcher in charge of the random grouping of the project (random center) of Shanghai University of Traditional Chinese Medicine, and only the person in charge of each clinical research center has the right to obtain the data.

All clinical research centers are responsible for the diagnosis and screening of clinical baseline data, and the physicians are responsible for recording and collecting the data.

The person in charge of each clinical research center shall arrange full-time data collection personnel to collect the intervention symptoms, signs, and follow-up data. They shall not actively know the random grouping, intervention plan, and other information.

Statistical analysis results data are collected and provided by the researcher in charge of data statistical analysis (statistical center) of Shanghai University of Traditional Chinese Medicine, who do not know the random grouping and clinical intervention information. After clinical trial completion, the personnel from the “statistical center” will go to each clinical research center to find the person in charge of each subcenter to coordinate and collect the case observation form, record the observation form in the database, and lock it under the supervision of the general research director of the project and the project supervisor (not directly involved in the project research).

##### Plans to promote participant retention and complete follow-up {18b}

To ensure retention of participants, follow-up visits will be scheduled to coincide with routine clinic appointments as far as possible. The study staff will contact participants, either over the phone or WeChat, before their scheduled follow-up appointment, immediately after the first treatment; 3 days and 1, 2, 4, 8, and 12 weeks after the intervention; and at 26 and 52 weeks after treatment follow-up.

##### Data management {19}

Before the start of the project, a complete project research operation SOP, investigator manual, and case report form should be established, and work training and summary meetings should be held before the start of the project and in the middle of the project, with detailed training and explanation of the test scheme, research operation SOP, and investigator manual contents. The case observation director of each research center should be established, and the case study work micro should be established by the person in charge of each research center credit group: case observation doctors in charge of each center should take photos of collected cases in the working group of each center, and give them to the person in charge of the subcenter who should, on the day of receipt, upload the standardized filing of the collected case report form. Consequently, Shuguang Hospital, the responsible unit of the project, should assign a special case supervisor, who should establish a WeChat working group with the person in charge of each center, and the supervisor should spot check the case report form from each center at any time to fill in the specification. After the test, the researcher (statistical center) in charge of project data statistics and analysis, a full-time statistician of Shanghai University of Traditional Chinese Medicine, should collect the original data from the case report form of each subcenter, and under the supervision of the chief research director of the project and the project supervisor (not directly involved in the project research), the assistant should cooperate with two people to back up and input those case reports into an Excel form, mutually proofread and correct input errors, check the accuracy with the original data in the case report form, lock the data, and then conduct statistical analysis.

##### Confidentiality {27}

The medical records (CRF, report form, etc.) of the participants will be completely saved in the research center of the project. All the privacy data of the subjects are stored in encrypted protection, only to be seen by the main researchers of the project, only for the research of the project, not for other purposes.

#### Plans for collection, laboratory evaluation, and storage of biological specimens for genetic or molecular analysis in this trial/future use {33}

Not applicable in this study.

#### Statistical methods

##### Statistical methods for primary and secondary outcomes {20a}

Statistical results and data analysis are the responsibility of the researcher (Statistics Center) in charge of statistics and data analysis for the entire project, and the patients are divided into the following data sets according to different situations.

Full analysis set refers to the ideal set of subjects as close as possible to the intentional analysis principle (mainly including all randomized subjects). The data set is obtained after the elimination of the smallest and reasonable method among all randomized subjects. To estimate the missing value of the main variable, the last observation carried forward (LOCF) method is used to carry forward the missing part of the test data. The number of subjects in each group who evaluate efficacy at the end point is consistent with that at the beginning of the test.

Per-protocol set, all patients who meet the trial protocol, who use 80–120% of drugs, have good compliance, do not use prohibited drugs during the trial, and complete the CRF requirements.

Safety analysis set, all cases are randomly divided into groups, at least one use of study drug, or receive manual treatment, and at least one follow-up record, constitute the safety analysis population of this study. Safety population is the main population for safety evaluation in this study.

The main variables and the comprehensive efficacy analysis are the full analysis set and the compliance scheme set, respectively; the demographic and other baseline characteristics are the full analysis set; the safety evaluation is the safety set.

For continuous variables, the number of cases, mean, standard deviation, median, and minimum and maximum values will be listed; for classified variables, the form of frequency table (frequency and percentage) will be listed. Baseline is defined as the last observation data before the first treatment.

Specific statistical data analysis, using the latest SPSS statistical analysis software, first of all, paired *t* test statistical analysis is carried out for each group’s pre-treatment pain and the improvement of each group’s pain after treatment intervention, and single-factor analysis of variance is carried out for each group’s immediate, 3-day, 1-, 2-, 4-, 12-week pain after intervention; secondly, before treatment and immediately after treatment intervention, 3 days, 1, 2, 4, and 8 weeks. One-way ANOVA is used to analyze the cervical dysfunction index, cervical activity, overall improvement, and treatment satisfaction of patients at 12 weeks. All the statistical tests are double-sided, *P* < 0.05 would be considered statistically significant.

##### Methods for additional analyses (e.g., subgroup analyses) {20b}

There will be no other additional analyses beyond the main analyses for the primary and secondary outcomes.

##### Methods in analysis to handle protocol non-adherence and any statistical methods to handle missing data {20c}

When the case falls off or the patient’s compliance is poor, the reasons for the fall off and poor compliance shall be entered in the case observation form in detail, and the patient’s understanding and support shall be obtained by contacting the patient as frequently as possible; the evaluation items that can be completed shall be completed, and the last treatment time shall be recorded. The case fall off, the patient’s compliance, and adverse reactions shall be statistically described and compared and evaluated between the groups.

##### Interim analyses {21b}

In the middle of the project, a complete project research operation SOP, investigator manual, and case report form should be reassessed and revised, and work training and summary meetings should be held with detailed training and explanation of the test scheme, research operation SOP, and investigator manual contents. A summary of the enrolment progress, treatment success proportions, adverse events, and protocol deviations will be provided to the Data Safety Monitoring Board members, who are not involved in experimental research and treatment.

##### Plans to give access to the full protocol, participant-level data, and statistical code {31c}

The protocol of the study is publicly available on the website of China Registered Clinical Trial Registration Center with no. ChiCTR1900021371.The datasets generated and/or analyzed during the current study are not publicly available due to China laws on privacy protection but are available from the corresponding author on reasonable request.

#### Oversight and monitoring

##### Composition of the coordinating center and trial steering committee {5d}

This project is under the direct supervision and management of the research team from Shanghai University of TCM. The research team and the special case inspector of Shuguang Hospital, the responsible unit of this project, have established a research supervision WeChat group. The supervisor of Shuguang Hospital and the person in charge of each center have established WeChat working group, and the supervisor can spot check the filling standard of case report form of each center at any time. The person in charge of each subcenter shall establish a WeChat group for the case study, and the case observation doctors of each center shall take real-time photos of the collected cases to the person in charge of each subcenter in the working group of each center on the same day and upload the standard situation of filling in the collected case report form.

##### Composition of the data monitoring committee, its role, and reporting structure {21a}

The Data and Safety Monitoring Board (DSMB) will be composed of a physician, medical statistician, ethicist, orthopedic doctor, radiologist, and clinical manager and will oversee the study throughout the study period. They will review the study activities every 3 months. The committee will review the safety data and clinical efficacy reports and determine whether it is clinically safe to continue the clinical trial. They will report their recommendation to the primary investigator from Shanghai University of TCM.

##### Adverse event reporting and harms {22}

During the test, we should observe whether there are adverse reactions and adverse events, and make records. If there are adverse events, such as adverse effects on patients’ consciousness, feeling, exercise, sleep, blood pressure, pulse, heart rate, respiration, and other normal physiological indicators, cervical fracture, skin damage, spinal cord and vertebral artery injury, gastrointestinal discomfort or ulcer, bleeding, and black stool, the occurrence of adverse reactions shall be recorded in detail on the case observation form, including the time, severity, duration, and treatment measures, and correlation with experimental treatment method shall be analyzed with comprehensive consideration of complications and combined treatment. In case of adverse reactions, the clinical observation physician can decide whether to stop the trial according to patient condition. Patients who stop treatment due to adverse reactions should be tracked and investigated, and the results should be recorded in detail.

##### Frequency and plans for auditing trial conduct {23}

This project is under the direct supervision and management of the research team from Shanghai University of TCM. The research team and the special case inspector of Shuguang Hospital, the responsible unit of this project, have established a research supervision WeChat group. The supervisor of Shuguang Hospital and the person in charge of each center have established WeChat working group, and the supervisor can spot check and audit the filling standard of case report form of each center at any time.

##### Plans for communicating important protocol amendments to relevant parties (e.g., trial participants, ethical committees) {25}

After careful discussion and modification by the project team, the study plan of this project was registered on the website of China Registered Clinical Trial Registration Center and further improved and modified according to the modification opinions provided by the expert team, with approval number ChiCTR1900021371. SOP and investigator’s manual for the research scheme of the project are formulated, and full-time researchers of each clinical research center are trained for the project. Each center is required to strictly follow the SOP and investigator’s manual for the research scheme. The ethics of this trial research scheme was reviewed and approved by China registered clinical trial ethics review committee with approval number ChiECRCT20190068. It was modified and implemented according to the expert’s modification opinions.

##### Dissemination plans {31a}

We plan to disseminate the study results through peer-reviewed journal publications and conference presentations. Study findings will also be shared with relevant clinical and scientific groups.

##### Drug distribution and recovery

The control group is responsible for the distribution of the trial drug by the treatment physician. The patient is only given a 1-week drug quantity when entering the group. The kit is recovered by the observer during the follow-up visit, and the drug application registration form is filled in according to the judgment of the drug application situation. When the amount reaches 80–100% of the required amount of drug, the next course of the drug can be given according to the pain relief situation if the patient needs to continue the treatment. At the end of each course of treatment, each patient needs to recover the kit, and fill in the daily and each course of treatment on the medication registration form.

## Discussion

The purpose of this study is to confirm our hypothesis through clinical trials that Shi’s cervical manipulation has more advantages in the efficacy, safety, and satisfaction of acute and subacute neck pain than traditional NSAIDs.

To achieve the goal of the study, a multicenter, positive-controlled, randomized clinical trial, comparing traditional analgesic drugs (NSAIDs) for neck pain is used to evaluate and show that Shi’s manipulation is more effective, safe, and satisfactory in the treatment of acute and subacute neck pain.

According to the latest survey [1–3], 71.5% of the general population had neck pain for more than a year. Although neck pain caused only 1.7–11.5% of disability, it is worth noting that, equivalent to back pain, it caused a huge burden and disability rate on individuals and the social economy and was listed as 1 of 5 chronic and refractory diseases by the US government. With the continuous development of social life, the high incidence rate of neck pain is not only confined to developed countries such as the USA, but also affects the developing countries, and has become a worldwide urgent disease [22–24]. At present, the clinical treatment of neck pain is no more than surgical treatment and non-surgical treatment [25]. Various clinical case studies confirm that the long-term effect advantage of surgical treatment is very limited compared with non-surgical treatment [26–29]. The latest research shows that clinical management of neck pain should pay close attention to the active diagnosis and treatment of acute and subacute neck pain, in an attempt to and thwart its progression to chronic neck pain [1, 16, 30]. Previous clinical trials have confirmed that Western chiropractic manipulation, health education, family exercise, and drugs are the main non-surgical treatment measures for acute and subacute cervical pain [11, 31–33]. Chiropractic manipulation, which can effectively relieve patients’ neck pain and cervical dysfunction, has both short- and long-term effects with clinical advantages, while drug treatment generally extends the treatment course. Therefore, clinicians should pay more attention to the cost of treatment, adverse drug reactions, and patient acceptance [11, 30, 34].

Tai Chi and bone-setting manipulation are the essence of TCM and have played an indelible role in China’s prevention and treatment of human diseases from thousands of years ago to modern times. In recent years, Tai Chi has been studied by foreign scholars, and a lot of clinical and experimental research has been invested in the application of Tai Chi in the treatment of bone and joint, insomnia, obesity, and other physical and mental diseases [35, 36]. Keyword searches for “neckpain,” “traditional Chinese medicine bone-setting manipulation,” and “acute subacute neck pain” in PubMed, Ovid, EMBASE, and MEDLINE foreign databases confirmed the global interest. The research in TCM bone-setting manipulation lags behind in the field of foreign medicine neck pain. As early as the Qing Dynasty, the manual treatment of neck pain was recorded in detail in the “Traditional Chinese Medical Orthopedics monograph” *Chinese Bone-setting Diagram*: “If the neck is injured, the head on the back cannot be lowered, or the tendon is long and bone is wrong, or the tendon is gathered, or the tendon is strong, use the second manipulation of *Xiong Gu Zi’s* technique to lift and correct the neck.”

On this basis, doctors developed many cervical vertebra techniques and formed different schools with different characteristics and positive curative effects in the diagnosis and treatment of neck pain. “Shi’s Traumatology,” a valuable cultural heritage passed from generation to generation in Shanghai over a hundred of years, is one of the most distinctive schools of “Shanghai culture.” And the cervical vertebra correction technique developed from it, “Dislocation of Bone and Malposition of Ligament,” is mature and effective. Shi’s cervical correction technology can reduce the recurrence rate, improve the efficiency of the price ratio, and support obvious clinical advantages in the diagnosis and treatment of neck pain [8–10]. However, compared with the first-NSAID treatment of neck pain, Shi’s cervical manipulation lacks the relevant research basis in terms of efficacy advantage, safety, and satisfaction in the treatment of neck pain.

With reference to the clinical trial design related to acute and subacute neck pain in this study, a multicenter positive-controlled, randomized clinical trial design is used [11, 14, 25, 37, 38]. Patients with acute and subacute neck pain in accordance with the test and research standards are randomly divided evenly into two groups: trial group (with Shi’s manipulation of the cervical spine as the main intervention method) and control group (with oral administration of diclofenac as the main intervention method), with both groups treated by corresponding main intervention method for up to 12 weeks. Clinical data were collected before the intervention and immediately after the first treatment; at 3 days and 1, 2, 4, 8, and 12 weeks after the intervention; and at 26 and 52 weeks after treatment follow-up of clinical observation index data collection. The clinical observation indices were as follows: (1) cervical pain is the primary observation index, measured by NRS; secondary indices include: (2) cervical dysfunction index, measured by patient self-evaluation using cervical NDI; (3) cervical activity measurement, measured by the cervical vertebra mobility measurement program of Android mobile phone system; (4) overall improvement, measured by patient self-evaluation with SF-36 (The Medical Outcomes Study 36-Item Short-Form Health Survey); and (5) satisfactory treatment, determined by patient self-evaluation, is 1 of 3 levels: dissatisfied, satisfied, and very satisfied.

Compared with some excellent multicenter clinical projects, this project has some limitations: for example, due to the limitation of research funds, this study only selected three clinical trial centers and small sample size of case observation, and only set up a control group, and only has 1 year follow-up period. Therefore, this study is only a preliminary exploration of the clinical evaluation scheme of “effectiveness and safety of Shi’s manipulation in the treatment of acute and subacute neck pain”. In order to further objectively and fully confirm the effectiveness and safety of Shi’s manipulation in the treatment of acute and subacute neck pain in the future, these research deficiencies will be supplemented and improved in our follow-up research.

### Trial status

The treatment protocol version number currently in use is version 1.0, which was revised on 15 October 2019. Recruitment began on 30 October 2019, and the approximate date for the completion of recruitment will be 31 December 2020.

## Abbreviations

LOCF: Last observation carried forward
NDI: Neck Disability Index
NRS: Numerical Rating Scale
NSAID: Non-steroidal anti-inflammatory drug
SF-36: The Medical Outcomes Study 36-Item Short-Form Health Survey
SOP: Standard operating procedures
TCM: Traditional Chinese medicine
3D CT: 3-Dimensional computed tomography
